# The Influence of Secular and Theological Education on Pastors’ Depression Intervention Decisions

**DOI:** 10.1007/s10943-013-9756-4

**Published:** 2013-07-12

**Authors:** Jennifer Shepard Payne

**Affiliations:** Jane Addams College of Social Work, University of Illinois at Chicago, 1040 West Harrison Street, Chicago, IL 60607-7134 USA

**Keywords:** Clergy, Depression, Pastors, Counseling, Treatment

## Abstract

Will a pastor refer to a mental health center? If they feel qualified to intervene themselves, they may not. Because pastors often provide grief counseling, it is important to understand the decisions they make when intervening with depressed individuals. A random sample of 204 Protestant pastors completed surveys about their treatment practices for depression. Fisher’s exact analyses revealed that more pastors with some secular education yet no degree felt that they were the best person to treat depression than pastors who had no secular education or pastors who had at least a secular bachelor’s degree. However, the level of theological education did not influence beliefs about the pastor being the best person to treat depression. In addition, neither secular nor theological education level influenced pastors’ views on referring people to mental health centers for depression treatment. Based on findings, this paper discusses implications for best practices in training pastors on depression and other mental health topics.

## Introduction

The National Comorbidity Survey-Replication study found that only 37 % of people with a depressive disorder get help within the first year of an episode. Approximately 88 % of people with major depressive disorder eventually make it into mental health treatment, but the average treatment delay for those who do not get help that first year can be from 6 to 8 years (Kessler et al. 2003; Wang et al. 2005).

Instead of traditional mental health centers, informal counseling supports are often the first assistance contact for depressed individuals (McCullough and Larson 1999; Neighbors et al. 1998; Stanford and Philpott 2011; Taylor et al. 2000). Studies have determined that individuals contact clergy more often for counseling than psychiatrists or general medical doctors (Wang et al. 2003; Young et al. 2003). There are several reasons why clergy are first responders to emotional concerns (Oppenheimer et al. 2004). While there is stigma involved in approaching a mental health professional for treatment, there is no stigma attached to talking with one’s own pastor (Runnels and Stauber 2011). Clergy consultations are often free, while traditional mental health centers provide services primarily through health insurance programs or managed care. Particularly in needy communities, distrust of the “system” is common (which can include distrust of traditional mental health centers), while a sense of trust and respect in community-based clergy exists (Milstein 2003; Payne 2008). This behavior of reliance on clergy for counseling occurs even more for Blacks and other ethnic groups (Chatters et al. 2011; Cooper et al. 2003; Levin 1986; Carrasco 2004; Taylor et al. 2011; Williams et al. 1999; Young et al. 2003).

Pastors (defined as clergy who are the heads of churches) have been intervening in the lives of depressed or suicidal congregants, families, and community members for generations (Ellison et al. 2006; VanderWaal et al. 2012; Weaver et al. 1997; Young et al. 2003). “Similar to primary care physicians, ministers are often the front-line responders in mental health, serving as natural helpers within a community as well as gatekeepers to more formal treatment”—p. 124 (Kramer et al. 2007). Pastors do encounter mental health problems on a regular basis. A study by Young et al. (2003) examined responses of 99 Black clergy and determined that two out of five pastors had severely mentally ill individuals in their congregations. Two out of three pastors reported involvement with suicidal individuals, and two out of five pastors spent more than 10 % of their counseling time performing crisis intervention. Besides religious/spiritual problems, grief issues (correlated with depression) were identified most often among the issues encountered (Young, et al. 2003). Because of the nature of their role, by default pastors must cope with a variety of psychological problems and mental health issues in their daily ministerial activities (Wasman et al. 1979).

Although pastors deal with these issues regularly, the literature suggests that clergy are not receiving educational experiences that equip them to handle these issues in a way that coincides with counseling methods promoted today (evidence-based practice); “clergy are largely unprepared to meet the mental health needs of parishioners” (Bledsoe and Adams 2011). For example, ministers in a study by Kramer and colleagues identified depression as one of the primary mental health problems in their congregations. Yet, many of the ministers in Kramer’s study lacked formal coursework, training, and experience (supervised training) in mental health issues. Ministers in the study were “concerned about their lack of training and expertise in diagnostic and treatment issues, conflicts between their pastoral and counseling roles, and feelings of being overwhelmed with the mental health concerns of their congregants (Kramer et al. 2007)”—p. 134. Moran and colleagues stated that additional specialized education for clergy is often a need, since “clergy see their seminary training as being only fairly adequate in preparing them to deal with grief, death and dying, and marital problems, and somewhat inadequate to preparing them to deal with depression, alcohol/drug problems, and suicide”(Moran et al. 2005)—p. 264.

Unfortunately, some of the published empirical literature that discusses clergy involvement in mental health alludes to certain untested assumptions. Some of these assumptions include the following:

- Most pastors are uneducated (or, at the very least, antiquated in their thinking).
- Those pastors with education primarily have theological educational backgrounds only.
- Clergy with a theological education (even with a graduate school education) are not equipped to make decisions about mental health in general and depression specifically:
  - 1. They do not understand when to refer someone out to a mental health center or a professional
  - 2. They do not realize that they should not be handling depressed cases
  - 3. They are more likely to be against a medical model of treatment than for it
- Pastors with secular education degrees are more likely to make referrals to mental health centers than those without secular degrees (especially if their secular degrees were in a health- or counseling-related field).

Researchers need to complete additional empirical studies in order to confirm or refute these assumptions.

There are some specialized educational experiences available for clergy. For instance, Moran and colleagues discuss Clinical Pastoral Education (CPE). Male pastors in their study with at least one unit of CPE training felt more competent in dealing with all types of issues than male pastors who had no CPE training (Moran et al. 2005). However, it appears that CPE training indeed remains an elite specialization; in Moran’s (2005) study, less than half of the pastors had some form of CPE training, largely due to financial and time constraints (Moran et al. 2005). Another potential specialization available to clergy includes pastoral counseling training through the American Association of Pastoral Counselors (AAPC). The AAPC defines pastoral counseling as the provision of “psychologically sound therapy that weaves in the religious and spiritual dimension” (AAPC 2009). Those pastors who have obtained certification through the AAPC have at least a bachelor’s degree from an accredited college or university, a 3-year professional degree from a seminary, and a specialized master’s or doctoral degree in the mental health field (AAPC 2009). Thus, those pastors who are certified as pastoral counselors under the AAPC umbrella have been exposed to detailed training on mental health issues as other non-clergy masters-level and doctoral-level counselors have been. Unfortunately, though, there are few active pastors who have been through the certification process with AAPC, and the numbers of certified pastors of color and pastors serving low-economic backgrounds are even lower (Paul 2005).

Furthermore, there are differences in the reported numbers of pastors who have received any type of pastoral counseling training (AAPC or otherwise) at all. While one report stated that 70 % of pastors they studied completed a pastoral care and counseling course in seminary (Vandecreek et al. 1998), in another study only 25 % of pastors surveyed had pastoral counseling training (Payne 2009). Again, pastors of color and pastors serving low-economic areas may have less access to pastoral counseling training opportunities (Augsburger 1989; Bruder 1965).

There remains great variation in the type of training that pastors receive before taking on leading a church congregation. There are a multiplicity of faiths, sects, denominations, and doctrines, and with this diversity, there are a range of seminary and bible college experiences. In addition, pastors have a variety of experiences in the secular college arena (“secular” is defined as traditional, non-religious education). Yet, few if any published studies have examined the impact of theological and secular educational experience on pastors’ depressive intervention decisions. This study is significant because it can inform best practices for addressing the mental health education needs of pastors who provide depression intervention.

This study explores how theological and/or secular education affects pastor’s views on how to intervene with depressed individuals. We use data from the Clergy Depressive Counseling Survey (Payne 2009) to examine the association between variables regarding best ways to treat depression and variables regarding secular or theological education level.

This research attempts to test some of the previously mentioned assumptions. The research hypothesis for this study is that if assumptions are correct, there should be a statistically significant difference in pastors’ decisions on whether to treat a depressed individual themselves or to refer out, based upon their levels of secular education. Based on the assumptions, it would be logical that the higher the level of secular education, the more likely the pastors will be open to referring to mental health centers. Based on those assumptions, there should also be a significant difference in pastors’ decisions based upon a pastor’s area of secular education (health- or counseling-related training compared with other secular training). A logical deduction based on assumptions would be that more counseling-related training would cause pastors to agree with mental health referral more readily. However, if no significant differences exist, or if other factors besides secular education influence differences, then there is a need for these untested assumptions to be re-examined.

This study also attempts to discover whether a pastor’s present theological or secular education level influences his or her desire for further training. Results from this study may inform best practices in training pastors on mental health topics such as depression.

## Methods

### Operational Definitions

The operational definition of the term “Pastor” in this study is a head of a church from one of 26 Protestant denominations. “Ministers” are licensed and/or ordained individuals licensed by an authoritative overseeing church body. “Clergy” is an all-inclusive term that includes both Protestant pastors and other ministers. “Race” in the study was defined by pastor’s self-report based on a list of close-ended options and one open-ended “other—please explain” option. This study used the variable “religious affiliation” instead of church denomination. Religious affiliation is defined based upon the definition of the term in the study by Meador et al. (1992). Each denomination fell under one of the following categories: Mainline Protestants, Conservative Protestants, Pentecostals, and Non-denominational (Payne 2009).

### Participants

The Clergy Depressive Counseling Survey is a survey of 204 Protestant English-speaking pastors and ministers from the State of California. Pastors and ministers who had either accessible e-mail addresses or church mailing addresses were included in the study. Protestant pastors from 26 denominations chose to participate.

### Procedure

The study used a proportional stratified random sampling plan to recruit participants. The procedures included the following:

1. An alphabetical list of 491 cities in California were randomized (via www.randomizer.org) and placed in numerical order based upon the randomization (Urbaniak and Plous 2013).
2. Using two online directories (www.Superpages.com and www.Switchboard.com), a comprehensive list of all church entries from the first California city on the list was created.
3. Researchers obtained a random sample of 10 % of the listed churches from that city.
4. Researchers repeated #2 and #3 for the second city on the list and continued this procedure for the subsequent cities listed until researchers obtained a sampling frame of approximately 1,000 churches.

Researchers used a mixed-mode survey strategy involving both e-mail and mail-out attempts at recruitment. E-mail recruitment was used as a first-choice strategy, and mailed letters were sent when e-mail was not available (Schaefer and Dillman 1998). Both of the online directories used included information about the church’s address, phone number, and church Web site when available. After obtaining a random sample, researchers divided the sample into two lists—churches with Web sites and churches without Web sites. Researchers explored each church Web site to obtain e-mail addresses for as many pastors as possible.

For pastors with e-mail addresses, pastors obtained an e-mailed letter of invitation to engage in the survey by computer (located at http://surveymonkey.com). E-mailed non-responders received a follow-up e-mail in 2 weeks. Pastors without e-mail addresses obtained written letters sent to their church address with a letter of invitation to participate in the study, an informed consent form, the survey itself, and a self-addressed stamped envelope. Mailed non-responders received one follow-up mailing to their church address 1 month after the initial mailing.

### Measures

Researchers designed the Clergy Depressive Counseling Survey questionnaire based on the data received from qualitative interviews with church leaders, pilot testing (three times), and adjustment based on feedback. Pastors responded to a survey instrument with 45 items (including demographic questions, questions on pastors’ views of the causes of depression and suicide, and questions about how pastors intervene in the lives of depressed and suicidal individuals). When asked about theological training and secular training, pastors completed Likert-scale questions regarding the subject, followed by open-ended questions. For instance, pastors answered questions about what level of secular training they had (no college, some college, Associate, Bachelors, Masters, or Doctorate degrees) and then filled out open-ended questions detailing their secular education. When asked about the best ways to counsel depressed individuals, pastors answered questions via a 5-point Likert scale with the following choices: Almost Always True, Usually True, Occasionally True, Usually Not True, and Almost Never True. The UCLA Office for the Protection of Research Subjects reviewed and approved all IRB requirements for this study, including pilot testing. Data collection began in July 2006 and concluded in August 2007.

### Sampling Plan

Researchers sent 1,126 invitations to participate in the study (344 e-mailed and 782 mailed); the pastors initially sampled were from 61 cities. From these contacts, researchers received 53 letters returned to sender, and 36 e-mails bounced back as no longer valid. Thus, 1,037 pastors were sampled, and 212 responses were obtained (111 web-survey responses and 101 mailed survey responses).

The overall response rate was 20 %. There was a lower response rate from mailed pastors of 14 % and a higher response from e-mailed pastors of 34 %. A limitation of the study is that there was a high non-response rate; 52 % of those e-mailed did not respond, and 84 % of those mailed did not respond. The average response rate for e-mail surveys with a single contact is 28.5 and 41 % for two contacts (Schaefer and Dillman 1998). Mailed surveys with one follow-up yield an average response rate of 30–35 % (Kaplowitz et al. 2004).

However, the response rates of this study might be within normal range for a population such as pastors, since there are high turnover rates of pastors over congregations. New pastors over churches are not reflected in Yellow Page listings, and church Web sites may not be updated frequently, so the survey may have been addressed to a pastor no longer over the congregation. (The “Discussion” section details the study’s limitations.) Researchers eliminated eight respondents from the analysis due to missing data, resulting in 204 analyzed results.

### Data Analysis

Pastors answered the question: What do they feel is the best way to treat depression? Researchers presented six statements regarding this question, and pastors chose to agree or disagree with each statement. Pastors originally answered based on a Likert scale of five items: Almost Always True (5), Usually True (4), Occasionally True (3), Usually Not True (2), and Almost Never True (1). Researchers recoded the data as a binomial distribution of Agree (3, 4, and 5) and Disagree (1 and 2) for the purposes of this analysis.

Descriptive statistics, Fisher’s exact tests, and Wald/LR analyses were generated with Stata Software, version 11 (StataCorp 2009). Researchers used Fisher’s exact test to examine the effects of theological or secular education on views about the best ways to treat depression (statistical significance is *p* > 0.05). Other variables such as gender, age, race, religious affiliation, and number of years in the ministry were controlled.

## Results

### Pastor Characteristics

Table 1 shows the demographics of the pastors in the study. Of the 204 responding pastors, 175 were male and 29 were female, ranging in age from 20 to over 65 years of age. The majority of pastors (50 %) were between 50 and 64 years of age, followed by 28 % of pastors who were between 35 and 49 years of age. Sixty-five percent of the sample consisted of White pastors (65 %), followed by Black pastors (25 %). The category “Other” included Asian, Hispanic, and other non-White pastors (10 %) due to low numbers. There was a limited response from Asian and Hispanic pastors due to the sampling decision to include English-speaking pastors only. Most pastors in the sample had quite a bit of experience in the ministry; 33 % of the sample had been in the ministry between 11 and 20 years, followed by 32 % of the sample that had between 21 and 30 years of ministry experience.

**Table 1:** Demographics of pastors

*N*	*N*	%
Gender		
Male	175	86
Female	29	14
Age		
20–34 years	15	7
35–49 years	57	28
50–64 years	102	50
65 years and up	30	15
Race		
Black	51	25
White	133	65
Other	20	10
Average neighborhood SES (based on zip code of church)		
Under 35,000 a year	51	25
31–45,000 a year	61	30
46–65,000 a year	40	20
Over 65,000 a year	52	25
Years of ministry		
1–10	23	11
11–20	67	33
21–30	65	32
31–40	34	17
Over 40	15	7
Licensure/ordination		
Licensed or ordained	83	41
Holds no ministry license	121	59
Protestant religious affiliation		
Mainline Protestants	60	29
Conservative Protestants	51	25
Pentecostals	56	27
Non-denominational	31	15
Other	6	3

*N* = 204

Table 2 shows the descriptive information regarding the secular and/or theological education experiences of the pastors. A large number of the pastors in the sample (82 %) had some level of secular college education. Fifty-six percent of the pastors had at least a bachelor’s degree from a secular university. Nine percent of the pastors had secular Ph.D.’s, followed by 14 % which had a master’s degree from a secular college or university. In addition, 96 % of the pastors had some type of theological training. Nineteen percent of the pastors had theological doctoral degrees, followed by 36 % that had a master’s degree in divinity or another theological discipline.

**Table 2:** Secular and theological education

	*N*	%
Secular degrees		
No secular education	37	18
Less than a bachelor’s degree	52	26
Bachelor’s degree	64	31
Graduate degree	51	25
Areas of pastor’s secular education		
No secular education	37	18
Counseling or behavioral science (psychology, social work, etc.)	36	18
Health related (medical, public health, etc.)	11	5
Other degrees (basic science, business, humanities, etc.)	120	59
Theological degrees		
No theological education	9	4
Some bible college, no degree	53	26
Theological bachelor’s degree	30	15
Theological graduate degree	112	55
Areas of pastor’s theological education		
No theological education	9	4
General theology (bible-based, Christian education, etc.)	130	64
Pastoral care/church leadership	35	17
Christian counseling/psychology	30	15
Formal pastoral counseling training		
Had training	50	25
No training	154	75

*N* = 204

The pastors’ areas of education were diverse. Pastors had received degrees in over 35 secular areas. (Areas included business related; counseling related; physical, behavioral, and social sciences; humanities; engineering; computer science; history; economics; education; English and other languages; communications; law; liberal arts; music; philosophy; physical education; political science; health care related; and urban planning). Most pastors had more than one type of theological training, which included training given to obtain a minister’s or ordination license, training in seminary, training in bible schools, chaplaincy training, priesthood training, and other types of theological training. Many pastors (69 %) held at least a BA in theology, Christian education, or pastoral care from a theological training institution. For data analysis purposes, researchers collapsed these areas into eight categories—four secular and four theological (see Table 2). Only one-fourth of the pastors surveyed (25 %) had pastoral counseling training.

### The Best Way to Treat Depression

Pastors answered the following overarching question: “What are the best ways to treat depression?” Pastors were able to provide their agreement or disagreement with six separate Likert-scale statements based on that question:

1. The pastor or minister is the best person to talk to.
2. The best treatment is to refer to a mental health center.
3. The best treatment is for them to see a medical doctor.
4. The best treatment is for them to work out their issues w/family and friends.
5. The best treatment is for them to read the Word of God or pray their way out of the situation.
6. The best treatment is for them to praise God and trust that things will be fine.

The first three questions pertain to research questions examined in this manuscript (whether pastors counsel depression themselves or refer out). Thus, this analysis focuses on questions one through three.

#### The Pastor as the Best Choice of Treatment for Depression

Table 3 shows information on the percentages of pastors who agreed that the pastor or minister is the best person to talk to when treating depression. Overall, 80 % of the pastors believed that there were times and occasions when it was appropriate for a pastor to treat depression. Only 20 % of the pastors believed that a pastor should not treat depression.

**Table 3:** Pastors’ agreement with the statement that they are the best person to treat depression (*n* = 163/204)

Research question: “The Pastor is the best person to treat depression”			
Predictor variable	Secular education level	% in agreement (agree/total *n*)^a^	Fisher’s exact
Level of secular training	No secular training	70.3 (26 out of 37)	0.032*
	Some college, less than BA/BS	92.3 (48 out of 52)	
	Secular bachelor’s degree	75.0 (48 out of 64)	
	Secular graduate degree	80.4 (41 out of 51)	
Level of theological training	No theological training	77.8 (7 out of 9)	0.902
	Bible college, less than BA	77.4 (41 out of 53)	
	Theological bachelor’s degree	80.0 (24 out of 30)	
	Graduate theological degree	81.3 (91 out of 112)	
Area of secular education	No secular degree	70.3 (26 out of 37)	0.130
	Counseling-related degree	72.2 (26 out of 36)	
	Applied science or health degree	90.9 (10 out of 11)	
	Has other degrees	84.2 (101 out of 120)	
Area of theological education	No theological training	77.8 (7 out of 9)	0.265
	Bible/Christian Ed./general Theo	81.5 (106 out of 130)	
	Pastoral care/church leadership	68.6 (24 out of 35)	
	Christian counseling/psychology	86.7 (26 out of 30)	
Had pastoral counseling training in the past	No pastoral counseling training	81.8 (126 out of 154)	0.230
	Has pastoral counseling training	74.0 (37 out of 50)	

^a^Each line of this table reports the percentage of pastors *under each education level* that are in agreement with the question asked. Thus, percentages do not total to 100 %

* *p* > 0.05 = statistical significance

When examining differences in how pastors answered this question based on the demographic variables obtained, there were no differences in this view based on SES, pastor’s race, religious affiliation, number of years in the ministry, or type of theological education or licenses. In addition, pastors who had formal pastoral counseling training did not statistically differ from pastors who had no formal pastoral counseling training when answering this question.

Although the type of theological training or type of theological licensure had no effect on how pastors answered this question, there were differences based on the type of secular education a pastor had. Primarily, pastors who had a few secular college classes but had not obtained a bachelor’s degree felt strongest about pastors being the ones to talk to about depression (92 %). Pastors who had no secular education at all were most cautious about being the ones to talk to (70 % agreed that pastors are the best choice). Interestingly, pastors with a secular BA or even a secular graduate degree were slightly more cautious about this question (75 and 80 % in agreement, respectively) than those pastors who had a little secular education but no degree [*χ* ^2^(3) Fisher’s exact = 8.0863, *p* = 0.032].

More male pastors (83 %) than female pastors (62 %) agreed that a person’s pastor is the best one to talk to about depressive issues [*χ* ^2^(1) Fisher’s exact = 6.6946, *p* = 0.02]. Age also influenced this answer; 93 % of pastors between the ages of 36 and 50 agreed that a person’s pastor is the best one to talk to, followed by 80 % of pastors under the age of 35 and 74 % of pastors over the age of 50 [*χ* ^2^(2) Fisher’s exact = 8.7061, *p* = 0.007].

#### Views About Referral to Mental Health Centers for Depression Treatment

Table 4 shows information on the percentages of pastors who agreed with the statement “The best way to treat depression is to refer to a mental health center.” Overall, 77 % of pastors felt that it was always, almost always, or occasionally appropriate to refer depressed people to a mental health center. In contrast, 23 % of pastors did not feel that referring to a mental health center was the best choice for a depressed person.

**Table 4:** Pastors’ agreement that the best treatment for depression is mental health referral (*n* = 157/204)

Research question: “The best treatment is to refer to a mental health center”			
Predictor variable	Secular education level	% in agreement (agree/total *n*)^a^	Fisher’s exact
Level of secular training	No secular training	67.6 (25 out of 37)	0.341
	Some college, less than BA/BS	78.8 (41 out of 52)	
	Secular bachelor’s degree	82.8 (53 out of 64)	
	Secular graduate degree	74.5 (38 out of 51)	
Level of theological training	No theological training	77.8 (7 out of 9)	0.210
	Bible college, less than BA	79.2 (42 out of 53)	
	Theological bachelor’s degree	90.0 (27 out of 30)	
	Graduate theological degree	72.3 (81 out of 112)	
Area of secular education	No secular degree	67.6 (25 out of 37)	0.513
	Counseling-related degree	77.8 (28 out of 36)	
	Applied science or health degree	81.8 (9 out of 11)	
	Has other degrees	79.2 (95 out of 120)	
Area of theological education	No theological training	77.8 (7 out of 9*)*	0.527
	Bible/Christian Ed./general Theo	80.0 (104 out of 130)	
	Pastoral care/church leadership	71.4 (25 out of 35)	
	Christian counseling/psychology	70.0 (21 out of 30)	
Had pastoral counseling training in the past	No pastoral counseling training	74.0 (114 out of 154)	0.086
	Has pastoral counseling training	86.0 (43 out of 50)	

^a^Each line of this table reports the percentage of pastors *under each education level* that are in agreement with the question asked. Thus, percentages do not total to 100 %

* *p* > 0.05 = statistical significance

There was a statistically significant difference in views about referring to mental health treatment on only one of the demographic variables examined. A relatively smaller percentage of pastors (71 %) who were heads of churches yet had no formal minister’s licensure agreed that referral to a mental health center was the best treatment. However, more pastors holding either minister’s or ordination licensure (86 %) agreed that mental health referral was the best way to treat depression [*χ* ^2^(1) Fisher’s exact = 5.8117, *p* = 0.018]. Interestingly, there were no other statistically significant differences based on theological education, secular education, or any other demographic.

#### Views About Treatment by Medical Doctors for Depression

Table 5 shows information on the percentages of pastors who agreed with the statement “The best way to treat depression is … for the person to see a medical doctor.” Pastors answered if it was best for a depressed person to see a medical doctor. Overall, 89 % of pastors agreed that a depressed person should see their medical professional (with 39 % of these pastors writing in a fill-in space “it depends on the situation”). Only 11 % of pastors did not agree that seeing a medical doctor was a good choice for a depressed person. There were no statistically significant differences in views about referring to mental health treatment based on any of the demographic variables obtained, including theological or secular education.

**Table 5:** Pastors’ agreement that going to see a medical doctor is the best depression treatment (*n* = 182/204)

Research question: “It is best for depressed people to see a medical doctor”			
Predictor variable	Secular education level	% in agreement (agree/total *n*)^a^	Fisher’s exact
Level of secular training	No secular training	83.8 (31 out of 37)	0.205
	Some college, less than BA/BS	86.5 (45 out of 52)	
	Secular bachelor’s degree	95.3 (61 out of 64)	
	Secular graduate degree	88.2 (45 out of 51)	
Level of theological training	No theological training	88.8 (8 out of 9)	0.222
	Bible college, less than BA	84.9 (45 out of 53)	
	Theological bachelor’s degree	83.3 (25 out of 30)	
	Graduate theological degree	92.9 (104 out of 112)	
Area of secular education	No secular degree	83.8 (31 out of 37)	0.056
	Counseling-related degree	100.0 (36 out of 36)	
	Applied science or health degree	90.9 (10 out of 11)	
	Has other degrees	87.5 (105 out of 120)	
Area of theological education	No theological training	88.9 (8 out of 9)	0.491
	Bible/Christian Ed./general Theo	90.0 (117 out of 130)	
	Pastoral care/church leadership	82.9 (29 out of 35)	
	Christian counseling/psychology	93.3 (28 out of 30)	
Had pastoral counseling training in the past	No pastoral counseling training	87.7 (135 out of 154)	0.296
	Has pastoral counseling training	94.0 (47 out of 50)	

^a^Each line of this table reports the percentage of pastors *under each education level* that are in agreement with the question asked. Thus, percentages do not total to 100 %

* *p* > 0.05 = statistical significance

### Desire for Further Training

Pastors discussed whether they would be interested in further training on how to handle depression issues if they had the resource available to them. Seventy-seven percent of the pastors surveyed stated that they would be interested in such training. It is interesting, though, that there were differences in the demographics of the pastors who were most interested in training. Pastors who were heads of congregations in zip codes serving communities of lower socioeconomic status were more often associated with the desire for further training than churches in higher-SES communities. A total of 84 % of pastors with churches in zip codes with medium income of below $35,000 and 85 % of pastors in areas with 36–$45,000 median income desired further training. In contrast, only 68 % of pastors serving areas with 46–$65,000 median income and 67 % of pastors with churches in areas where individuals had a median income of over $65,000 a year desired further training [*χ* ^2^(3) Fisher’s exact = 8.6685, *p* = 0.034]. In addition, Black pastors (90 %) and pastors from other minority groups (90 %) were more often associated with the desire for further training than White pastors in this sample (70 %) [*χ* ^2^(3) Fisher’s exact = 10.6696, *p* = 0.004].

Unexpectedly, more pastors who had pastoral counseling training in the past also had a desire for further training on depression (88 %), compared with pastors who had no pastoral counseling training background (73 %) [*χ* ^2^(1) Fisher’s exact = 4.5522, *p* = 0.034].

## Discussion

This study investigated pastors’ theological and secular educational experiences to determine whether they influenced their decisions when intervening in the lives of depressed individuals. Only 25 % of the pastors surveyed had been involved in specific pastoral counseling training programs. Nevertheless, this sample of pastors was highly educated; pastors had a wide range of both secular and theological educational experiences. A large number of the pastors in the sample (82 %) had some level of secular college education, and 96 % of the pastors had some type of theological training.

There were only certain circumstances in which the pastors’ educational experiences influenced their thoughts on the best way to treat depression. Pastors who had a few secular college classes under their belt but had not obtained a bachelor’s degree felt strongest about the pastor being the best choice to treat depression, while pastors without any secular college experience and pastors who had at least a secular bachelor’s degree were more cautious in making this statement. More pastors who were licensed or ordained felt that referral to a mental health center was the best treatment than pastors who had no formal licensure.

There were some instances where additional factors besides education influenced pastor’s decisions about the best way to treat depression. More male pastors agreed that a person’s pastor is the best one to talk to about depressive issues. More mid-aged pastors (age 36–50) believed that a person’s pastor is the best one to talk to about depression than younger or older pastors. No religious affiliation differences were evident in any of the data discussed.

There is a poem by Alexander Pope that says, “a little learning is a dangerous thing.” Pastors who had a little secular college but no degree felt most confident that they are the best person to treat depression than other groups. A small amount of knowledge can mislead people into thinking that they are more expert than they really are. The findings influence practice implications: Very short training programs for pastors that give them a little mental health knowledge may give a false sense of security about counseling ability. This is important, because there is a present movement to train laity and clergy alike through a Mental Health First Aid training model, which trains individuals on spotting signs and symptoms of mental illness during a 2–3-day training module (Kitchener and Jorm 2002). More controlled empirical studies need to be done with pastors (control groups compared with treatment groups) to determine whether short trainings or conferences increase false confidence in clergy who handle mental health issues.

This study found that pastors who were unlicensed heads of churches were less likely to agree about referral to mental health centers than licensed pastors were. Unlicensed pastors are less likely to be a part of a State-authorized overseeing body such as a Council, a Religious Order, or any other church credentialing body. The fact that unlicensed pastors are more likely to be heads of independent churches (and they may work in isolation) may influence their decisions about referral.

It is interesting to note that certain statistically significant differences were notably absent. There were no differences in views about any type of referral based on secular or theological education level or area. Thus, educational training did not influence the decision to refer to a mental health center or a physician. As stated in the introduction, this research attempted to test some of the previously mentioned assumptions about clergy that are in the literature. Based on these assumptions, pastors with higher levels of secular education should be more open to referring to outside professionals, and pastors with more counseling-related training should more readily agree with mental health referral. However, this study found no significant differences in referral decisions based on education at all. Thus, the field needs to engage in more research to test assumptions about clergy presented in the literature.

Over three-fourths of pastors surveyed stated that they would be interested in further training on how to handle depression issues if presented with the opportunity. More pastors heading congregations in lower-SES neighborhoods and more minority pastors were interested than White pastors or those in higher-SES neighborhoods. Nine out of ten Black pastors and pastors from other minority groups desired further training.

There is a correlation between being a Black pastor and serving lower-SES neighborhoods. Mollica and colleagues found that few of the traditional and evangelical clergy and few of the pastoral counselors in their study counseled poor individuals, while many of the Black clergy did (Mollica et al. 1986). And similar to this study, Aten and colleagues found that a majority of Black pastors they surveyed reported a need and readiness for education and training targeting mental-health-related issues (Aten et al. 2011). Researchers have found that wealthier, resource-rich congregations are more likely to have organized community health ministries, while smaller, poorer congregations possess fewer resources (Catanzaro et al. 2007). Thus, Black pastors in particular have expressed an openness to consider more mental health training to deal with issues that they often face when servicing high-poverty areas (Conley and Wolfe 2011; Rowland and Isaac-Savage 2013; Stansbury 2011).

## Limitations and Strengths

Limitations of this study include its reliance on self-report data possibly affected by recall bias, self-selection, or demand characteristics. In addition, this is a cross-sectional survey, and as such, it is unable to capture changes in treatment practices over time. Finally, because of the low response rate, the data cannot generalize to all pastors.

The strength of this study, however, is the fact that it is the first of its kind to examine the specific theological and secular education characteristics of a sample of pastors and to determine whether their educational profiles influenced their depression treatment views.

## Conclusion and Implications

Based on the results of this study, it is important to develop models for training pastors that will cater to clergy from all socioeconomic backgrounds and cultures (Queener and Martin 2001; Wallace et al. 2012). The traditional educational formats that mental health professionals use (such as continuing education units or short certificate programs) may not be the best fit for pastors. When developing a conceptual framework for a mental illness training program for pastors, we need to deconstruct the assumptions we make as traditional clinicians (the medical model, psychological nomenclature, etc.). We also need to tailor the curriculum to the pastor’s role in the clinical setting (Holinger 1979). From past research, the majority of ministers are dissatisfied with the traditional workshop, weekend seminar, or conference method of learning because they offer information without any opportunity to develop skills. Instead, pastors want knowledge that directly applies to the day-to-day problems they face (Wasman et al. 1979).

A viable training program for pastors must have several characteristics. It must be flexible enough to serve the needs of pastors at varying levels of experience and formal training. It must accommodate to varying religious and cultural viewpoints (Allen et al. 2010). It must be able to serve inner city, suburban, and rural congregations from a variety of geographic locations. It has to accommodate diverse socioeconomic, racial, and ethnic compositions. Yet, it must also have the capacity to unify pastors on common themes, to create community.

## References

[CR1] AAPC. (2009). What is pastoral counseling? (The American Association of Pastoral Counselors) Retrieved November 28, 2011 from http://aapc.org/content/what-pastoral-counseling.

[CR2] Allen A, Davey M, Davey A (2010). Being examples to the flock: The role of church leaders and African American families seeking mental health care services. Contemporary Family Therapy.

[CR3] Aten JD, Topping S, Denney RM, Hosey JM (2011). Helping African American clergy and churches address minority disaster mental health disparities: Training needs, model, and example. Psychology of Religion and Spirituality.

[CR4] Augsburger, D. W. (1989). Pastoral counseling: Global anabaptist mennonite encyclopedia online. Retrieved November 28, 2011 from http://www.gameo.org/encyclopedia/contents/P381ME.html.

[CR5] Bledsoe, S., & Adams, C. (2011). *Promoting emotional well*-*being among southern California parishioners through clergy/mental health practitioner collaboration*. Paper presented at the North American Association of Christians in Social Work Annual Convention, Pittsburgh, PA.

[CR6] Bruder EE (1965). An overview of clinical pastoral education. Pastoral Psychology.

[CR23] Carrasco, M. (2004). NAMI African American outreach resource manual. Retrieved from http://www.nami.org/Content/ContentGroups/Multicultural_Support1/Fact_Sheets1/Outreach_Manuals/A_A_Resource_Manual.pdf.

[CR7] Catanzaro AM, Meador KG, Koenig HG, Kuchibhatla M, Clipp EC (2007). Congregational health ministries: A national study of pastors’ views. Public Health Nursing.

[CR8] Chatters LM, Mattis JS, Woodward AT, Taylor RJ, Neighbors HW, Grayman NA (2011). Use of ministers for a serious personal problem among African Americans: Findings from the National Survey of American Life. American Journal of Orthopsychiatry.

[CR9] Conley, C. M., & Wolfe, M. L. (2011). *An exploratory study of how African American clergy conceptualize mental health disorders and the utilization of mental health services*. MSW Thesis, California State University, Sacramento, Sacramento California. Retrieved from http://csus-dspace.calstate.edu/bitstream/handle/10211.9/1150/Thesis.pdf?sequence=1.

[CR10] Cooper LA, Gonzales JJ, Gallo JJ, Rost KM, Meredith LS, Rubenstein LV, Ford DE (2003). The acceptability of treatment for depression among African–American, Hispanic, and White primary care patients. Medical Care.

[CR11] Ellison CG, Vaaler ML, Flannelly KJ, Weaver AJ (2006). The clergy as a source of mental health assistance: What Americans believe. Review of Religious Research.

[CR12] Holinger PC (1979). The severely emotionally distressed: A conceptual framework and potential roles for clergy and church. Pastoral Psychology.

[CR13] Kaplowitz MD, Hadlock TD, Levine R (2004). A comparison of web and mail survey response rates. Public Opinion Quarterly.

[CR14] Kessler RC, Berglund P, Demler O, Jin R, Koretz D, Merikangas KR, Wang PS (2003). The epidemiology of major depressive disorder: Results from the National Comorbidity Survey Replication (NCS-R). JAMA.

[CR15] Kitchener BA, Jorm AF (2002). Mental health first aid training for the public: Evaluation of effects on knowledge, attitudes and helping behavior. BMC Psychiatry.

[CR16] Kramer TL, Blevins D, Miller TL, Phillips MM, Davis V, Burris B (2007). Ministers’ perceptions of depression: A model to understand and improve care. Journal of Religion and Health.

[CR17] Levin JS (1986). Roles for the Black pastor in preventive medicine. Pastoral Psychology.

[CR18] McCullough ME, Larson DB (1999). Religion and depression: A review of the literature. Twin Research.

[CR19] Meador KG, Koenig HG, Hughes DC, Blazer DG, Turnbull J, George LK (1992). Religious affiliation and major depression. Hospital & Community Psychiatry.

[CR20] Milstein, G. (2003). Clergy and psychiatrists: Opportunities for expert dialogue. *Psychiatric Times, 20*(3), 2. Retrieved from UBM Medica website: http://www.psychiatrictimes.com/display/article/10168/47231?page.

[CR21] Mollica RF, Streets FJ, Boscarino J, Redlich FC (1986). A community study of formal pastoral counseling activities of the clergy. The American Journal of Psychiatry.

[CR22] Moran M, Flannelly KJ, Weaver AJ, Overvold JA, Hess W, Wilson JC (2005). A study of pastoral care, referral, and consultation practices among clergy in four settings in the New York City area. Pastoral Psychology.

[CR24] Neighbors HW, Musick MA, Williams DR (1998). The African American minister as a source of help for serious personal crises: Bridge or barrier to mental health care?. Health Education & Behavior.

[CR25] Oppenheimer JE, Flannelly KJ, Weaver AJ (2004). A comparative analysis of the psychological literature on collaboration between clergy and mental-health professionals—perspectives from secular and religious journals: 1970–1999. Pastoral Psychology.

[CR26] Paul, P. (2005). With God as my shrink. *Psychology Today*. Retrieved 11-28-11, 2005 from http://www.psychologytoday.com/articles/200505/god-my-shrink.

[CR27] Payne JS (2008). “Saints Don’t Cry”: Exploring messages surrounding depression and mental health treatment as expressed by African–American Pentecostal preachers. Journal of African American Studies.

[CR28] Payne JS (2009). Variations in pastors’ perceptions of the etiology of depression by race and religious affiliation. Community Mental Health Journal.

[CR29] Queener JE, Martin JK (2001). Providing culturally relevant mental health services: Collaboration between psychology and the African American church. Journal of Black Psychology.

[CR30] Rowland, M. L., & Isaac-Savage, E. P. (2013). As i see it: A study of African American pastors’ views on health and health education in the Black church. *Journal of Religion and Health*. doi:10.1007/s10943-013-9705-2. 10.1007/s10943-013-9705-2 23563927

[CR31] Runnels, R. C., & Stauber, M. (2011). Today’s best pastoral care: Church-based mental health and social programs. *The Church Leader’s Counseling Resource Book: A Guide to Mental Health and Social Problems*, 431.

[CR32] Schaefer, D. R., & Dillman, D. A. (1998). Development of a standard e-mail methodology: Results of an experiment. *Public opinion quarterly,**62*(3), 378–397.

[CR33] Stanford M, Philpott D (2011). Baptist senior pastors’ knowledge and perceptions of mental illness. Mental Health, Religion & Culture.

[CR34] Stansbury KL (2011). Men of the cloth: African-American clergy’s knowledge and experience in providing pastoral care to African-American elders with late-life depression. Journal of Ethnic and Cultural Diversity in Social Work.

[CR35] StataCorp (2009). Stata statistical software: Release 11.

[CR36] Taylor RJ, Ellison CG, Chatters LM, Levin JS, Lincoln KD (2000). Mental health services in faith communities: The role of clergy in Black churches. Social Work.

[CR37] Taylor, R., Woodward, A., Chatters, L., Mattis, J., & Jackson, J. (2011). Seeking help from clergy among black Caribbeans in the United States. *Race and Social Problems* 1–11. doi:10.1007/s12552-011-9056-0.

[CR38] Urbaniak, G. C., & Plous, S. (2013). *Research randomizer* (Version 4.0) [Computer software]. Retrieved July 7, 2013 from http://www.randomizer.org/.

[CR39] Vandecreek L, Carl D, Parker D, Koenig HG (1998). The role of nonparish clergy in the mental health system. Handbook of religion and mental health.

[CR40] VanderWaal CJ, Hernandez EI, Sandman AR (2012). The gatekeepers: Involvement of Christian clergy in referrals and collaboration with Christian social workers and other helping professionals. Social Work and Christianity.

[CR41] Wallace AC, Proeschold-Bell RJ, LeGrand S, James J, Swift R, Toole D, Toth M (2012). Health programming for clergy: An overview of protestant programs in the United States. Pastoral Psychology.

[CR42] Wang PS, Berglund PA, Kessler RC (2003). Patterns and correlates of contacting clergy for mental disorders in the United States. Health Services Research.

[CR43] Wang PS, Berglund P, Olfson M, Pincus HA, Wells KB, Kessler RC (2005). Failure and delay in initial treatment contact after first onset of mental disorders in the national comorbidity survey replication. Archives of General Psychiatry.

[CR44] Wasman M, Corradi RB, Clemens NA (1979). In-depth continuing education for clergy in mental health: Ten years of a large scale program. Pastoral Psychology.

[CR45] Weaver AJ, Koenig HG, Larson DB (1997). Marriage and family therapists and the clergy: A need for clinical collaboration, training, and research. Journal of Marital and Family Therapy.

[CR46] Williams DR, Griffith EEH, Young JL, Collins C, Dodson J (1999). Structure and provision of services in Black churches in New Haven, Connecticut. Cultural Diversity and Ethnic Minority Psychology.

[CR47] Young JL, Griffith EEH, Williams DR (2003). The integral role of pastoral counseling by African-American clergy in community mental health. Psychiatric Services.

